# Perpetration of intimate partner violence and suicide attempt, suicidal ideation, and non-suicidal self-harm: a cross-sectional secondary analysis using the Adult Psychiatric Morbidity Survey

**DOI:** 10.1017/S2045796026100559

**Published:** 2026-03-26

**Authors:** Sophie Carlisle, Rachel Whyte, Katherine Saunders, Sally McManus, Sian Oram, Louise Howard, Vishal Bhavsar

**Affiliations:** 1 Institute of Psychiatry, Psychology and Neuroscience, King’s College London, London, UK; 2 Health Innovation East Midlands, Nottingham, UK; 3 Centre for Behaviour Change, University College London, London, UK; 4 Violence and Society Centre, City St George’s, University of London, London, UK

**Keywords:** forensic mental health, intimate partner violence, mental health services, non-suicidal self-harm, substance use, suicide

## Abstract

**Aims:**

Intimate partner violence (IPV) victimization is associated with suicidal behaviour. Suicidal behaviour may also be raised among those who perpetrate IPV compared to those who do not; general population-based evidence is, however, lacking. We aimed to investigate the associations between using violence against an intimate partner with suicidal thoughts, suicide attempt and non-suicidal self-harm in the past year.

**Methods:**

We analysed data from the 2014 Adult Psychiatric Morbidity Survey. Logistic regressions estimated associations between IPV perpetration and suicide attempt, suicidal ideation, and self-harm. Associations were estimated for men and women separately, and we explored interaction in estimates by IPV victimization.

**Results:**

After adjustment for demographic and socioeconomic covariates, lifetime IPV perpetration was strongly associated with past-year suicide attempt (men: odds ratio [OR] 3.6, 95% confidence interval 1.0–13.2, women: OR 4.2, 1.9–9.4), suicidal ideation (men: OR 2.7, 1.5–4.9, women: OR 2.6, 1.7–4.1) and self-harm (men: OR 4.9, 1.5–15.2, women: OR 3.3, 1.8–6.0). Estimates were substantially attenuated with adjustment for non-IPV life adversities, hazardous alcohol use, drug use and IPV victimization. Only the association with lifetime suicide attempt in women remained significant (OR 1.6, 1.1–2.3). Estimates were generally higher among those who had not experienced IPV victimization, although we found no evidence for interaction by IPV victimization on the association between IPV perpetration and suicidal behaviour.

**Conclusions:**

There were greater odds of suicidality and self-harm among self-reported perpetrators of IPV compared to the general population. Many of these associations were accounted for by non-IPV life adversities, IPV victimization and substance use. Improving the identification and management of IPV perpetration, and developing targeted safety planning and interventions for this group could reduce suicide for perpetrators and victims of IPV.

## Introduction

Suicide is a global public health problem and causes significant economic and social costs, including distress to friends and family (Cerel *et al.*, 2016; Samaritans, 2024). More than 700,000 people worldwide die by suicide annually (World Health Organization, 2021), of which three-quarters are men (World Health Organization, 2016; Office for National Statistics, 2021). In the UK specifically, over 7000 suicide deaths were registered in 2023 (Kirk-Wade, 2025), representing the highest suicide rate since 1999 (Office for National Statistics, 2024a), with over 76,000 hospital admissions related to self-harm in the same year (NHS England, 2024).

Intimate partner violence (IPV) is defined as any behaviour perpetrated by a current or former intimate partner that causes physical, sexual or psychological harm, including controlling behaviours (Heise and Garcia-Moreno, 2002; Council of Europe, 2014). In the year ending March 2024, the police in England and Wales recorded over 1.3 million domestic abuse-related incidents, of which 851,062 were recorded as crimes, leading to 51,183 prosecutions (Office for National Statistics, 2024b), although the actual prevalence of domestic abuse is likely much higher given that much goes unreported (Gracia, 2004; National Centre for Domestic Violence, 2022). Experiencing IPV is associated with suicide attempts (Seedat *et al.*, 2005; Ellsberg *et al.*, 2008; Cavanaugh *et al.*, 2011; Guillén *et al.*, 2015; McManus *et al.*, 2022), suicide ideation (Weaver *et al.*, 2007; Ellsberg *et al.*, 2008; Yanqiu *et al.*, 2011) and non-suicidal self-harming behaviours (Levesque *et al.*, 2010; Blosnich and Bossarte, 2012). Thus, IPV has been identified as relevant for suicide prevention in England’s National Suicide Prevention Strategy (Department of Health and Social Care, 2023).

Less attention has been paid to the relevance of IPV perpetration for suicide prevention. There is limited research on any association between perpetration of IPV and suicide in the general population. Evidence comes instead from selected populations, including people in contact with the criminal justice system, or engaged in behaviour change programmes or substance use treatment services. These studies suggest an association between perpetration of physical IPV and suicidal ideation in people using substance use treatment (Ilgen *et al.*, 2009), and between dating violence perpetration and suicidal ideation in young people (Nahapetyan *et al.*, 2014). Recent evidence from England suggests that men engaged in court-mandated behaviour change programmes for IPV perpetration experienced a nearly 30-fold increase in suicide rate (Conner *et al.*, 2002; Knipe *et al.*, 2024). However, these study populations are unlikely to be representative of the wider population. For example, those in contact with the criminal justice system may have poorer mental health, or have a higher suicide risk compared to those who are not (Webb *et al.*, 2011; Bebbington *et al.*, 2021), limiting the conclusions that can be drawn for population-based suicide prevention. Evidence from general population samples could be helpful in developing population-wide strategies to prevent suicide.

The association between IPV perpetration and suicidality is plausible because they may share common risk factors, for example, separate evidence links both childhood trauma to the perpetration of IPV (Li *et al.*, 2020) and to suicide (Zatti *et al.*, 2017). Suicidal behaviour and IPV perpetration may also share structural drivers, including masculine attitudes, which may involve finding violence more acceptable, and a greater reluctance to seek help when in distress (King *et al.*, 2020). This association is further supported by theoretical frameworks. The interpersonal-psychological theory of suicide (Joiner, 2005) proposes that the concurrent presence of two interpersonal states, thwarted belongingness (i.e., feelings of loneliness or isolation) and perceived burdensomeness (i.e., feelings of liability or worthlessness), predicts suicide ideation, while suicide death also requires acquired capability (i.e., fearlessness and pain tolerance), which may develop after repeated exposure to painful events. All of these factors may be elevated in both those who experience IPV as victims and those who perpetrate IPV. For instance, in those who perpetrate IPV, thwarted belongingness may be exacerbated by negative impacts on social relationships as a result of violent behaviours and their legal consequences, which may in turn generate feelings of self-hatred. Increased suicide capability has also been linked to aggression (Smith *et al.*, 2016), which in turn is associated with IPV perpetration (Cascardi *et al.*, 2018).

It is suggested that co-occurring IPV perpetration/victimization (i.e., the experience of both the victimization and perpetration of IPV by the same individual), also referred to as bidirectional IPV, has distinct mechanisms and impact from IPV perpetrated without co-occurring IPV victimization (Johnson and Leone, 2005), although this is debated (e.g., see Walby and Towers, 2018). Whether the relationship between IPV perpetration and suicide is influenced by the co-occurrence of IPV victimization is unknown.

There are clear gender differences across both suicidality and IPV victimization and perpetration. For example, men are up to four times more likely to die by suicide despite women being more likely to attempt suicide, in part due to differences in the lethality of methods chosen (Mergl *et al.*, 2015). On the other hand, women are more likely to engage in non-suicidal self-harm (Bresin and Schoenleber, 2015). Women are also more likely to be victims of all types of domestic abuse than men (Office for National Statistics, 2024c), while perpetrators are more likely to be male (Hester, 2013).

We examined the association between perpetration of IPV and suicide attempt, suicidal ideation, and non-suicidal self-harm in representative general population data from England. Our objectives were to:

1. Estimate the association of lifetime IPV perpetration with past-year suicide attempt, suicidal ideation and non-suicidal self-harm in men and women.
2. Explore heterogeneity in estimates based on the presence of IPV victimization.

## Methods

### Data collection

The Adult Psychiatric Morbidity Survey (APMS) is a cross-sectional, probability-sample survey with the most recent available data collected in 2014. Full details of the design can be found elsewhere (McManus *et al.*, 2020). Briefly, the survey sampled the household residential population of England aged 16 and above, using a stratified, multistage random sampling design, based on the national Small User Postcode Address File. The study sample comprised 7546 individuals, representing a response rate of 57%. Data collection was conducted primarily through computer-assisted personal interviewing in people’s homes by trained interviewers, with some items collected during a computer-assisted self-completion interview (CASI) section of the interview, in which the participant used the interviewer’s laptop.

### Measurements

#### IPV perpetration

Lifetime IPV perpetration was indicated if any of the following four items asked in the CASI were endorsed: ‘have you ever pushed, held or pinned down or slapped a partner or ex-partner?’; ‘have you ever kicked, bit, hit with a fist or something else, or thrown something at a partner or ex-partner that hurt them?’; ‘have you ever forced a partner or ex-partner to do something sexual that they didn’t want to do?’; and ‘have you ever frightened a partner or ex-partner, by threatening to hurt them or someone close to them?’. The wording of these items was adapted from the Short Form Conflict Tactics Scale (CTS2-S), which corresponds well to the full Conflict Tactics Scale (CTS; Straus, 1979), with good concurrent and construct validity (Straus and Douglas, 2004). Dichotomous (yes/no) variables were created for the lifetime experience of each type of IPV perpetration (threats, pushing, kicking and forcing your partner to do something sexual), any lifetime IPV perpetration and the number of types of IPV perpetration.

#### Suicide attempt, suicidal ideation and non-suicidal self-harm

Information on past-year suicide attempt was collected using the items: ‘have you ever made an attempt to take your life by taking an overdose of tablets or in some other way?’; and ‘was this in the last year?’; asked both in the face-to-face interview and the self-completion questionnaire, to give individuals more opportunities to disclose. Our variable measuring past-year suicide attempt was based on endorsement of either of these items. Lifetime suicide attempt was based on the item: ‘have you ever made an attempt to take your life by taking an overdose of tablets or in some other way?’, endorsed either face-to-face or during self-completion. Past-year suicidal ideation was measured based on the items ‘have you ever thought of taking your life, even if you would not really do it?’ and ‘was this in the past year?’, which were administered face-to-face only. Non-suicidal self-harm in the past year was measured based on the items ‘have you ever deliberately harmed yourself in any way but not with the intention of killing yourself?’ and ‘was this in the past year?’, endorsed either face-to-face or during self-completion. Dichotomous (yes/no) variables were created for past-year experience of suicide attempt, suicidal ideation and non-suicidal self-harm, and for lifetime experience of suicide attempt.

#### Lifetime experience of IPV

Items measuring lifetime experience of IPV were adapted from those included in the Crime Survey for England and Wales (Office for National Statistics, 2015), which is originally based on the CTS, a measure of IPV which has evidence of high reliability, with a median alpha coefficient of reliability of 0.86 across 10 studies (Straus and Mickey, 2012). One dichotomous (yes/no) variable was created for lifetime experience of IPV, based on endorsement of any of the following items: ‘experience of a partner preventing you from having a fair share of the household money’; ‘repeatedly belittling you to the extent that you felt worthless’; ‘pushing you, holding you, pinning you down or slapping’; ‘sending you more than one unwanted letter, email, text message or card that was either obscene or threatening and which caused you fear, alarm or distress’; or ‘kicking you, biting you, hitting you with a fist or something else, or throwing something at you that hurt you’.

#### Sociodemographic characteristics

Sociodemographic characteristics were self-reported by participants and included gender, age, educational attainment, marital status and ethnicity. Neighbourhood deprivation was measured according to the English Indices of Deprivation 2010 (Her Majesty’s Department of Communities and Local Government, 2011) and grouped into quintiles. Socioeconomic class was measured using the National Statistics Socio-Economic Classification (Leete and Fox, 1977). All sociodemographic characteristics were treated as categorical variables.

#### Other life adversities

To assess the number of lifetime adversities experienced other than IPV, a scale was created using the List of Threatening Experiences scale (LTE; Brugha *et al.*, 1985; McManus *et al.*, 2022), described as a binary variable cut at the median (0–2 vs. 3 or more), and handled as a continuous variable for modelling. Lifetime adversities included: serious illness or injury, serious illness or injury to a close relative, serious assault of a close relative, death of an immediate family member, death of a close family friend or other relative, violence at work, homelessness, redundancy or being sacked from a job, extended work search without success, major financial crisis, something valued being lost or stolen, having trouble with the police involving court appearance and serving time in prison. While psychometric validation of this adapted version of the LTE scale is unavailable, the original version shows good concurrent validity (sensitivity of 0.89–1.0 and specificity of 0.74–0.88), and test–retest reliability in all but one item (Cohen’s kappa 0.78–1.0) (Brugha and Cragg, 1990).

#### Hazardous alcohol use

Hazardous alcohol use was determined using the 10-item Alcohol Use Disorders Identification Test (AUDIT-10) scale (Saunders *et al.*, 1993). A score of 8 or above indicated hazardous (including harmful or dependent) alcohol use. The AUDIT-10 has high diagnostic validity, with a sensitivity of 0.92 and specificity of 0.94 (Saunders *et al.*, 1993), and high reliability with an average Cronbach’s α of 0.8 over 10 studies (de Meneses-gaya *et al.*, 2009). This outcome was treated as a dichotomous variable, with responses categorized as ‘yes’ (where an individual scored 8 or above) or ‘no’.

#### Drug use in the past year

Measurement of drug use in the past year was based on any use of cannabis, amphetamines, cocaine, crack, ecstasy, heroin, crystal methamphetamine, tranquilizers, amyl nitrate/poppers, anabolic steroids, glue/solvents/gas/aerosols, acid/LSD and magic mushrooms in the past year. This was treated as a dichotomous variable, with responses categorized as ‘yes’ (where responses indicated the use of any of the aforementioned drugs) or ‘no’.

### Analysis

**Figure 1.**
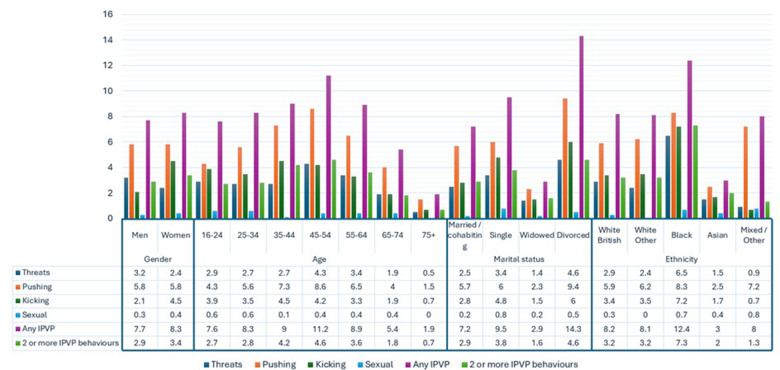
Diagram displaying the distribution of IPV perpetration by sociodemographic characteristics.

Analyses were done in Stata 17 (StataCorp, 2022) and were based on 6462 participants with complete data on the study variables. We described any IPV perpetration, two or more IPV perpetration behaviours, the four IPV perpetration behaviours separately and lifetime experience of IPV victimization (Table 1 and Figure 1), and past-year suicide attempt, lifetime suicide attempt, past-year suicidal ideation and past-year non-suicidal self-harm (Table 2) according to sociodemographic and other covariates, using counts and survey-weighted percentages. We present a complete case analysis, with respondents with complete data defined as those with observed data on all exposure, outcome and covariate variables described in Table 1. Inference assumes that data were missing completely at random, that is, that the probability of being missing for each data point is independent of both observed and unobserved data.

**Table 1.** Characteristics of the sample reporting lifetime IPV perpetration behaviours and indicators for non-partner violence and IPV perpetration. Proportions (%) are weighted for the study design

		Lifetime IPV perpetration behaviours													
	Number in row category	Threats		Pushing		Kicking		Sexual		Any IPVP		Two or more IPV perpetration behaviours		Lifetime experience of IPV^a^	
	*N*	*N*	%	*N*	%	*N*	%	*N*	%	*N*	%	*N*	%	*N*	%
**Gender**															
**Men**	2568	78	3.2	157	5.8	60	2.1	6	0.3	202	7.7	77	2.9	417	15.6
**Women**	3894	105	2.4	233	5.8	185	4.5	14	0.4	345	8.3	134	3.4	1149	27.2
**Age**															
**16–24**	436	16	2.9	24	4.3	21	3.9	3	0.6	40	7.6	16	2.7	113	22.1
**25–34**	916	29	2.7	60	5.6	42	3.5	5	0.6	88	8.3	32	2.8	265	24.2
**35–44**	1049	28	2.7	77	7.3	52	4.5	1	0.1	101	9.0	43	4.2	310	26.8
**45–54**	1163	49	4.3	98	8.6	57	4.2	5	0.4	136	11.2	53	4.6	359	24.8
**55–64**	1055	35	3.4	75	6.5	42	3.3	3	0.4	105	8.9	39	3.6	276	23.0
**65–74**	1031	22	1.9	46	4.0	25	1.9	3	0.4	63	5.4	22	1.8	165	13.4
**75+**	812	4	0.5	10	1.5	6	0.7	0	0.0	14	1.9	6	0.7	78	9.1
**Marital status**															
**Married/cohabiting**	3753	89	2.5	201	5.7	103	2.8	8	0.2	262	7.2	103	2.9	721	18.9
**Single**	1203	45	3.4	92	6.0	75	4.8	9	0.8	138	9.5	57	3.8	354	23.6
**Widowed**	666	10	1.4	17	2.3	12	1.5	1	0.2	22	2.9	12	1.6	86	12.1
**Divorced**	840	39	4.6	80	9.4	55	6.0	2	0.5	125	14.3	39	4.6	405	46.2
**Ethnicity**															
**White British**	5565	159	2.9	342	5.9	213	3.4	17	0.3	482	8.2	180	3.2	1380	22.5
**White Other**	354	7	2.4	19	6.2	11	3.5	0	0.0	25	8.1	10	3.2	66	17.3
**Black**	160	10	6.5	12	8.3	12	7.2	1	0.7	19	12.4	11	7.3	42	22.6
**Asian**	264	5	1.5	9	2.5	7	1.7	1	0.4	12	3.0	7	2.0	48	14.7
**Mixed/Other**	119	2	0.9	8	7.2	2	0.7	1	0.8	9	8.0	3	1.3	30	21.0
**Tenure**															
**Owner-occupier**	4359	79	2.0	204	4.8	103	2.1	6	0.2	275	6.3	88	2.1	827	17.4
**Social renter**	984	60	5.7	93	8.3	75	6.7	8	0.9	139	12.6	68	6.5	368	32.4
**Private renter**	119	44	3.6	93	7.4	67	5.0	6	0.5	133	10.5	55	4.3	371	28.1
**Employment**															
**Employed**	3605	106	3.1	255	6.8	160	3.9	11	0.4	348	9.1	139	3.7	969	23.4
**Unemployed**	178	10	4.3	17	7.0	8	3.2	3	1.1	27	11.7	6	2.3	68	32.7
**Inactive**	2679	67	2.2	118	4.0	77	2.5	6	0.2	172	5.8	66	2.3	529	17.6
**IMD quintiles (area deprivation)**															
**1 (least deprived)**	1377	27	1.9	67	5.0	35	2.2	1	0.1	91	6.3	29	2.1	264	17.9
**2**	1356	19	1.6	61	4.3	34	2.2	2	0.2	84	5.7	25	1.9	280	18.2
**3**	1355	36	2.6	71	5.2	43	3.1	4	0.5	101	7.6	41	3.1	326	21.7
**4**	1220	44	3.9	89	7.4	55	4.5	7	0.7	119	9.8	56	4.8	340	24.5
**5 (most deprived)**	1154	57	4.2	102	7.5	78	5.2	6	0.3	152	11.0	60	4.2	356	26.4
**Hazardous alcohol use^b^**															
**No**	5329	128	2.2	266	4.8	170	2.8	14	0.3	376	6.5	145	2.6	1232	20.5
**Yes**	1133	55	5.1	124	10.1	75	5.7	6	0.6	171	14.2	66	5.3	334	26.2
**Drug use in past year^c^**															
**No**	6038	146	2.4	319	5.2	196	3.0	13	0.2	447	7.0	165	2.7	1374	20.2
**Yes**	424	37	7.2	71	13.0	49	7.9	7	1.8	100	18.8	46	8.2	192	37.3
**Other life adversities^d^**															
**Low (less than 3)**	2828	52	1.7	99	3.3	58	1.8	8	0.3	151	5.0	54	1.7	519	16.2
**High (3 or more)**	3634	131	3.8	291	8.1	187	4.8	12	0.4	396	10.7	157	4.5	1047	26.5
**Column totals**	**6462**	**183**	**2.8**	**390**	**5.8**	**245**	**3.4**	**20**	**0.3**	**547**	**8.0**	**211**	**3.2**	**1566**	**21.7**

a Experience of IPV was measured based on items for: ‘experience of a partner preventing you from having a fair share of the household money’; ‘repeatedly belittling you to the extent that you felt worthless’; ‘pushing you – holding you – pinning you down or slapping’; ‘sending you more than one unwanted letter – email – text message or card that was either obscene or threatening and which caused you fear – alarm or distress’; or ‘kicking you – biting you – hitting you with a fist or something else – or throwing something at you that hurt you’.

b Indicated by a score of 8 or above on the AUDIT-10 scale.

c Based on any use of cannabis – amphetamines – cocaine – crack – ecstasy – heroin – crystal methamphetamine – tranquilizers – amyl nitrate/poppers – anabolic steroids – glue/solvents/gas/aerosols – acid/LSD – and magic mushrooms in the past year.

d Lifetime adversities included experience of: serious illness or injury – serious illness or injury to a close relative – serious assault of a close relative – death of an immediate family member – death of a close family friend or other relative – violence at work – homelessness – redundancy or being sacked from a job – extended work search without success – major financial crisis – something valued being lost or stolen – having trouble with the police involving court appearance – and serving time in prison.

**Table 2.** Characteristics of the sample reporting past-year and lifetime suicide attempts – past-year suicidal ideation and past-year non-suicidal self-harm

	Number in row category	Suicide attempts (past-year)		Suicide attempts (lifetime)		Suicidal ideation (past-year)		Self-harm (past-year)	
	*N*	*N*	%	*N*	%	*N*	%	*N*	%
**Gender**									
**Men**	2568	18	0.9	131	5.2	115	4.6	24	1.2
**Women**	3894	38	0.9	323	7.8	185	4.4	73	2.2
**Age**									
**16–24**	436	19	3.9	49	9.8	39	8.3	38	7.7
**25–34**	916	10	1.0	87	8.3	49	5.6	20	1.9
**35–44**	1049	8	0.6	88	7.5	59	4.3	17	1.2
**45–54**	1163	6	0.4	97	6.4	63	4.8	14	0.8
**55–64**	1055	11	0.5	82	6.7	59	4.1	6	0.4
**65–74**	1031	2	0.1	42	3.3	20	1.5	1	0.1
**75+**	812	0	0.0	9	1.4	11	1.5	1	0.1
**Marital status**									
**Married/cohabiting**	3753	8	0.2	158	4.2	97	2.8	20	0.6
**Single**	1203	31	3.0	170	12.5	110	8.9	58	5.6
**Widowed**	666	3	0.4	16	2.2	25	3.3	2	0.2
**Divorced**	840	14	1.3	110	12.7	68	7.3	17	1.9
**Ethnicity**									
**White British**	5565	47	0.8	395	6.4	261	4.6	84	1.6
**White Other**	354	2	1.0	17	5.4	14	4.0	5	1.8
**Black**	160	4	3.2	13	8.9	7	5.0	3	3.0
**Asian**	264	2	1.7	18	7.8	10	3.9	5	2.6
**Mixed/Other**	119	1	0.5	11	7.1	8	4.4	0	0.0
**Tenure**									
**Owner-occupier**	4359	11	0.4	175	3.9	129	3.3	22	0.7
**Social renter**	984	28	2.7	159	14.7	89	7.9	34	3.7
**Private renter**	119	17	1.3	120	9.5	82	6.1	41	3.8
**Employment**									
**Employed**	3605	16	0.6	225	5.7	144	4.0	41	1.3
**Unemployed**	178	3	1.0	24	10.1	25	13.0	8	3.6
**Inactive**	2679	37	1.5	205	7.7	131	4.6	48	2.3
**IMD quintiles (area deprivation)**									
**1 (least deprived)**	1377	4	0.4	48	3.7	31	2.2	12	0.1
**2**	1356	10	0.8	58	4.0	61	4.5	17	1.8
**3**	1355	12	1.1	88	6.1	61	4.6	24	2.2
**4**	1220	9	0.7	106	7.4	64	4.9	16	1.4
**5 (most deprived)**	1154	21	1.6	154	12.0	83	6.3	28	2.2
**Hazardous alcohol use**									
**No**	5329	34	0.7	336	5.9	200	3.5	69	1.4
**Yes**	1133	22	1.8	118	9.1	100	8.5	28	2.9
**Drug use in the past year**									
**No**	6038	35	0.5	356	5.3	230	3.5	62	1.1
**Yes**	424	21	5.0	98	20.4	70	14.9	35	8.4
**Other life adversities**									
**Low**	2828	17	0.7	111	3.5	91	3.4	34	1.4
**High**	3634	39	1.1	343	9.3	209	5.5	63	2.0
**Lifetime experience of IPV**									
**No**	4896	23	0.6	190	3.9	150	3.4	38	1.1
**Yes**	1566	33	2.0	264	16.0	150	8.5	59	3.9
**Column totals**	6462	56	0.9	454	6.5	300	4.5	97	1.7

To test objective 1, we estimated survey-weighted logistic regression models including a multiplicative interaction term (IT) for gender in all models *a priori*, to allow gender-disaggregated estimates to be reported by applying a linear combination to estimate odds ratios (ORs) with 95% confidence intervals (95% CIs) (Howard *et al.*, 2017). We selected covariates for adjustment based on the shared risk factors for IPV perpetration (Moore *et al.*, 2007; Foran and O’Leary, 2008; Clare *et al.*, 2021; Spencer *et al.*, 2022) and suicide (Masango *et al.*, 2008; DeBastiani *et al.*, 2019; Park *et al.*, 2020; Favril *et al.*, 2022). These were age, ethnicity, marital status, tenure, area deprivation, other life adversities, hazardous alcohol use, drug use in the past year and experience of IPV. We estimated associations between indicators of IPV perpetration, which were threats towards your partner; pushing your partner; kicking your partner; forcing your partner to do something sexual; any lifetime IPV perpetration; and the count of distinct IPV perpetration behaviours, with each outcome (past-year suicide attempt; lifetime suicide attempt; past-year suicidal ideation; and past-year self-harm). To explore the impact of covariate adjustment on associations, we report partially adjusted models including only age, ethnicity, marital status, tenure and area deprivation (model 1), adding other life adversities (model 2), further adding hazardous alcohol use and drug use in the past year (model 3) and finally including the experience of IPV in the lifetime (model 4). For any lifetime IPV perpetration only, we report IT estimates with 95% CIs, and Wald test *p*-values, to assess interaction by gender for past-year and lifetime suicide attempt, past-year suicidal ideation, and past-year self-harm.

To test objective 2, we estimated multiplicative ITs for the experience of IPV, presenting estimates for those with and without lifetime experience of IPV. All covariates were entered into models, and partially adjusted estimates were reported (models including only age, ethnicity, marital status, tenure and area deprivation defined as model 1, adding other life adversities to estimate model 2, further adding hazardous alcohol use and drug use in the past year to estimate model 3). For any lifetime IPV perpetration only, we reported IT estimates with 95% CIs, and Wald test *p*-values to assess interaction by IPV exposure for past-year and lifetime suicide attempt, past-year suicidal ideation, and past-year self-harm.

## Results

### Description of the study population

A total of 6462 participants provided complete data on variables included in this analysis, of whom 2568 were men, and 3894 were women. The prevalence of reporting any lifetime IPV perpetration was 7.7% in men and 8.3% in women (Table 1 and Figure 1). In terms of specific IPV perpetration behaviours, the prevalence of reporting threats was 3.2% in men and 2.4% in women. The prevalence of reporting pushing was 5.8% in both men and women. For kicking, the prevalence was 2.1% in men and 4.5% in women, and for sexual violence, the prevalence was 0.3% in men and 0.4% in women. Lifetime IPV perpetration was most commonly reported by participants aged 45–54 years (11.2%), and least commonly by those aged 75 years and older (1.9%). The prevalence of IPV perpetration was 7.2% among married/cohabiting participants, 9.5% among single participants, 2.9% among widowed participants and 14.3% among divorced participants. The prevalence of any reported lifetime IPV perpetration was 8.2% among White British participants, 8.1% among White Other participants and 8.0% among Mixed/Other participants. The proportion of Black participants reporting lifetime IPV perpetration was 12.4%, while the proportion for Asian participants was 3.0%.

The prevalence of suicide attempt in the past year was the same in men and women (0.9%), and the prevalence of lifetime suicide attempt was 5.2% in men and 7.8% in women (Table 2). Prevalence of past-year suicidal ideation in men and women was similar (4.6% men; 4.4% women). The prevalence of past-year non-suicidal self-harm was 2.2% in women and 1.2% in men. The prevalence of suicide attempts (past-year and lifetime), suicidal ideation and non-suicidal self-harm, reduced with increasing age of participants (e.g., comparing 16–24-year-olds with 75-year-olds and above: past-year suicide attempt: 3.9% vs. 0.0%; lifetime suicide attempt: 9.8% vs. 1.4%; past-year suicidal ideation: 8.3% vs. 1.5%; and past-year self-harm: 7.7% vs. 0.1%). Compared to other groups based on marital status, single participants had the highest prevalence of past-year suicide attempt (3.0%), past-year suicidal ideation (8.9%) and past-year self-harm (5.6%). The prevalence of lifetime suicide attempt was highest among divorced participants (12.7%). Black participants experienced a greater prevalence of past-year and lifetime suicide attempts (3.2% and 8.9%, respectively), past-year suicidal ideation (5.0%), and past-year self-harm (3.0%), compared to other ethnic groups.

### Associations of IPV perpetration with suicide attempt, suicidal ideation and non-suicidal self-harm

For any lifetime IPV perpetration, model 1 estimates were statistically significant for all comparisons (Table 3). After all adjustments (model 4), associations were attenuated, and the only comparison remaining statistically significant was between lifetime IPV perpetration and lifetime suicide attempt in women (OR 1.6, 95% CI 1.1–2.3).

**Table 3.** Associations (odds ratios – ORs – with 95% CIs in parentheses) of IPV perpetration behaviours with suicide attempt – suicidality and non-suicidal self-harm in men and women. All models based on 6462 records with complete data on modelled variables

IPV perpetration behaviour	Suicidality outcome		Model 1	Model 2	Model 3	Model 4
Lifetime IPV perpetration	Suicide attempt (past-year)	Men	**3.6 (1.0–13.2)**	2.1 (0.5**–**8.7)	1.5 (0.4**–**6.0)	1.3 (0.3**–**5.6)
		Women	**4.2 (1.9–9.4)**	**2.8 (1.1–6.6)**	2.3 (0.9–5.6)	1.9 (0.7–4.8)^a^
	Suicide attempt (lifetime)	Men	**2.6 (1.5**–**4.5)**	1.5 (0.8–2.8)	1.3 (0.7–2.4)	0.9 (0.5–1.8)
		Women	**3.5 (2.5**–**4.8)**	**2.5 (1.8**–**3.6)**	**2.4 (1.7**–**3.4)**	**1.6 (1.1**–**2.3)^b^**
	Suicidal ideation (past-year)	Men	**2.7 (1.5**–**4.9)**	**1.9 (1.0**–**3.6)**	1.5 (0.8–3.0)	1.3 (0.6–2.7)
		Women	**2.6 (1.7**–**4.1)**	**2.0 (1.3**–**3.2)**	**1.8 (1.1**–**2.8)**	1.5 (0.9–2.4)^c^
	Non-suicidal self-harm (past-year)	Men	**4.9 (1.5**–**15.2)**	2.8 (0.9–9.2)	2.3 (0.7–7.6)	1.9 (0.5–6.6)
		Women	**3.3 (1.8**–**6.0)**	**2.2 (1.2**–**4.1)**	**1.9 (1.0**–**3.5)**	1.5 (0.7–3.1)^d^
Number of distinct IPV perpetration behaviours	Suicide attempt (past-year)	Men	**2.6 (1.4**–**4.9)**	**2.0 (1.1**–**3.7)**	1.0 (0.5–2.0)	0.9 (0.4–1.9)
		Women	**1.8 (1.3**–**2.6)**	**1.4 (1.0**–**2.1)**	1.4 (0.9–2.2)	1.3 (0.8–2.0)
	Suicide attempt (lifetime)	Men	**1.8 (1.3**–**2.3)**	1.3 (0.9–1.8)	1.1 (0.8–1.6)	0.9 (0.6–1.4)
		Women	**1.9 (1.6**–**2.2)**	**1.6 (1.3**–**1.9)**	**1.5 (1.2**–**1.8)**	**1.3 (1.0**–**1.5)**
	Suicidal ideation (past-year)	Men	**2.1 (1.6**–**2.8)**	**1.7 (1.2**–**2.4)**	1.3 (0.9–2.0)	1.2 (0.8–1.9)
		Women	**1.7 (1.4**–**2.1)**	**1.5 (1.2**–**1.8)**	**1.3 (1.1**–**1.7)**	**1.2 (1.0**–**1.5)**
	Non-suicidal self-harm (past-year)	Men	**2.2 (1.2**–**3.8)**	1.6 (0.8–3.0)	1.6 (0.8–3.3)	1.5 (0.7–3.0)
		Women	**1.8 (1.4**–**2.4)**	**1.5 (1.1**–**2.0)**	**1.4 (1.0**–**1.9)**	1.3 (0.9–1.8)
Threat	Suicide attempt (past-year)	Men	3.8 (0.6–25.1)	2.5 (0.3–18.1)	1.8 (0.3–12.0)	1.7 (0.2–12.1)
		Women	**6.2 (2.2**–**17.5)**	**3.6 (1.3**–**10.5)**	**3.7 (1.3**–**10.4)**	**3.0 (1.0**–**8.8)**
	Suicide attempt (lifetime)	Men	1.9 (0.8–4.4)	1.1 (0.4–2.9)	0.9 (0.4–2.2)	0.8 (0.3–2.0)
		Women	**5.5 (3.2**–**9.4)**	**4.0 (2.2**–**7.3)**	**4.1 (2.2**–**7.4)**	**2.7 (1.5**–**5.0)**
	Suicidal ideation (past-year)	Men	**2.8 (1.1**–**7.8)**	2.0 (0.7–5.4)	1.5 (0.5–4.4)	1.4 (0.5–4.0)
		Women	**4.5 (2.5**–**8.3)**	**3.3 (1.8**–**6.3)**	**3.3 (1.8**–**6.3)**	**2.8 (1.5**–**5.3)**
	Non-suicidal self-harm (past-year)	Men	**5.4 (1.2**–**23.4)**	3.6 (0.8–16.4)	2.8 (0.6–12.7)	2.6 (0.6–12.0)
		Women	**5.3 (2.4**–**11.7)**	3.2 (0.6–12.7)	**3.1 (1.5**–**6.6)**	**2.4 (1.1**–**5.2)**
Pushing	Suicide attempt (past-year)	Men	**3.0 (1.0**–**9.7)**	1.6 (0.5–5.0)	1.1 (0.3–3.9)	0.9 (0.2–3.0)
		Women	**2.9 (1.2**–**7.5)**	1.6 (0.6–4.3)	1.4 (0.5–3.8)	1.1 (0.4–3.1)
	Suicide attempt (lifetime)	Men	**2.4 (1.3**–**4.5)**	1.3 (0.7–2.6)	1.1 (0.5–2.2)	0.7 (0.4–1.6)
		Women	**2.6 (1.8**–**3.9)**	**1.8 (1.2**–**2.7)**	**1.6 (1.1**–**2.5)**	1.1 (0.7–1.6)
	Suicidal ideation (past-year)	Men	**3.0 (1.5**–**5.8)**	**2.0 (1.0**–**4.1)**	1.6 (0.7–3.4)	1.3 (0.6–3.0)
		Women	**2.3 (1.3**–**3.8)**	1.6 (0.9–2.8)	1.5 (0.9–2.6)	1.2 (0.7–2.2)
	Non-suicidal self-harm (past-year)	Men	3.2 (0.8–13.0)	1.6 (0.4–7.0)	1.3 (0.3–6.0)	0.9 (0.2–4.6)
		Women	**2.7 (1.3**–**5.6)**	1.5 (0.7–3.3)	1.4 (0.6–3.1)	1.0 (0.5–2.4)
Kicking	Suicide attempt (past-year)	Men	0.9 (0.1–7.2)	0.3 (0.0–2.7)	0.2 (0.0–2.3)	0.2 (0.0–1.9)
		Women	**3.2 (1.2**–**8.6)**	1.6 (0.6–4.7)	1.3 (0.4–3.8)	1.0 (0.4–3.1)
	Suicide attempt (lifetime)	Men	**4.0 (1.7**–**9.7)**	2.2 (0.8–6.0)	2.1 (0.7–6.0)	1.6 (0.6–4.4)
		Women	**4.1 (2.8**–**6.0)**	**2.6 (1.7**–**4.1)**	**2.4 (1.6**–**3.7)**	**1.7 (1.1**–**2.6)**
	Suicidal ideation (past-year)	Men	**4.0 (1.6**–**9.8)**	2.6 (0.9–7.2)	2.2 (0.7–6.6)	1.9 (0.6–5.8)
		Women	**2.5 (1.5**–**4.1)**	**1.7 (1.0**–**2.8)**	1.5 (0.8–2.5)	1.2 (0.7–2.1)
	Non-suicidal self-harm (past-year)	Men	4.2 (0.7–23.8)	1.8 (0.3–9.6)	1.7 (0.3–11.2)	1.4 (0.2–8.1)
		Women	**3.7 (1.8**–**7.6)**	**2.1 (1.0**–**4.6)**	1.7 (0.8–3.6)	1.4 (0.6–3.0)
Forcing your partner to do something sexual	Suicide attempt (past-year)	Men	–	–	–	–
		Women	–	–	–	–
	Suicide attempt (lifetime)	Men	–	–	–	–
		Women	–	–	–	–
	Suicidal ideation (past-year)	Men	–	–	–	–
		Women	–	–	–	–
	Non-suicidal self-harm (past-year)	Men	**33.9 (1.2**–**954.5)**	**33.2 (1.7**–**659.2)**	21.5 (0.7–642.4)	17.8 (0.7–455.2)
		Women	**5.5 (1.0**–**29.6)**	**5.6 (1.1**–**29.6)**	3.9 (0.8–18.7)	4.2 (0.8–21.0)

*Note*: Model 1: adjusted for age – ethnicity – marital status – tenure – and area deprivation. Model 2: adjusted for model 1 variables – and also for other life adversities. Model 3: adjusted for model 2 variables – and also for hazardous alcohol use – and drug use in the past year. Model 4: adjusted for model 3 variables and also for experiencing IPV in the lifetime. Bold text indicates statistical significance.

a Gender IT: 1.5 (95% CI 0.3–7.2) – *p* = 0.628.

b Gender IT: 1.7 (95% CI 0.9–3.4) – *p* = 0.118.

c Gender IT: 1.1 (95% CI 0.5–2.5) – *p* = 0.763.

d Gender IT: 0.8 (95% CI 0.8–2.9) – *p* = 0.728.

Similarly, model 1 estimates for the number of IPV perpetration behaviours were statistically significant for all comparisons and displayed attenuation on adjustment for covariates, with lifetime suicide attempt in women (OR 1.3, 1.0–1.5), and past-year suicidal ideation in women (OR 1.2, 1.2–1.5) remaining statistically significant among model 4 estimates.

For threats, estimates adjusted only for sociodemographic and socioeconomic variables (model 1) were statistically significant except for past-year suicide attempt, and lifetime suicide attempt, in men. After all adjustments (model 4), ORs remained statistically significant for past-year suicide attempt in women (OR 3.0, 1.0–8.8), lifetime suicide attempt in women (OR 2.7, 1.5–5.0), past-year suicidal ideation in women (OR 2.8, 1.5–5.3) and past-year self-harm in women (OR 2.4, 1.1–5.2).

For pushing, estimates from model 1 were statistically significant except for past-year non-suicidal self-harm in men. After all adjustments, no estimates remained statistically significant for this outcome in either men or women.

For kicking, OR estimates for model 1 were large (between 2.5 and 4.2 across variables and gender), except for past-year suicide attempt in men, where the OR was 0.9 (0.1–7.2). Other estimates were also statistically significant except for past-year non-suicidal self-harm in men. After all adjustments (model 4), estimates remained statistically significant for lifetime suicide attempt in women only (OR 1.7, 1.1–2.6).

For sexual IPV, models for suicide attempt and suicidal ideation did not produce stable estimates owing to sparse strata and are not reported. For past-year non-suicidal self-harm, the model 1 estimate for men was large in magnitude (OR 33.9, 1.2–954.5), and smaller for women (OR 5.5, 1.0–29.6). After adjustment for all covariates, these estimates were substantially attenuated and no longer statistically significant. CIs, particularly for men, were very wide (men: OR 17.8, 0.7–455.2; women: OR 4.2, 0.8–21.0), indicating strongly that these estimates should be interpreted with caution and that the role of chance cannot be ruled out.

Tests for interaction by gender in the association of any lifetime IPV perpetration with suicide attempt, suicidal ideation and non-suicidal self-harm were non-significant (past-year suicide attempt IT: 1.5 [0.3–7.2], *p* = 0.628; lifetime suicide attempt IT: 1.7 [0.9–3.4], *p* = 0.118; past-year suicidal ideation IT: 1.1 [0.5–2.5], *p* = 0.763; and past-year non-suicidal self-harm IT: 0.8 [0.8–2.9], *p* = 0.728).

On stratification by IPV victimization (Table 4), all estimates were greater in magnitude for those without IPV victimization compared to those with IPV victimization for threats, pushing and kicking, any IPV, and the number of IPV behaviours. In contrast, for sexual IPV perpetration, estimates were greater in magnitude for those with IPV victimization compared to those without IPV victimization in relation to past-year and lifetime suicide attempt, although final estimates were unstable due to small numbers with the outcome. After all adjustments, only the association of any IPV perpetration with past-year suicidal ideation in those without IPV victimization (OR 2.6, 1.2–5.9), and past-year non-suicidal self-harm in those without IPV victimization (OR 3.8, 1.2–11.9) remained statistically significant. Tests for interaction by IPV victimization in the association of any lifetime IPV perpetration with suicide attempt, suicidal ideation and non-suicidal self-harm were also non-significant (past-year suicide attempt IT: 0.6 [0.1, 4.2], *p* = 0.604; lifetime suicide attempt IT: 0.6 [0.3–1.4], *p* = 0.217; past-year suicidal ideation IT: 0.4 [0.2–1.0], *p* = 0.059; and past-year non-suicidal self-harm IT: 0.3 [0.1–1.2], *p* = 0.081).

**Table 4.** Associations (odds ratios – ORs – with 95% CIs in parentheses) of IPV perpetration behaviours with suicide attempt (past year and lifetime) – suicidal ideation in the past year – and self-harm in the past year – stratified by IPV exposure. All models based on 6462 records with complete data on modelled variables

IPV perpetration behaviour	Suicidality outcome		Model 1	Model 2	Model 3
Any IPV behaviour	Suicide attempt (past-year)	No IPV exposure	4.9 (0.9–27.0)	3.4 (0.6–21.1)	2.4 (0.4–14.0)
		IPV exposure	**2.2 (1.0**–**4.9)**	1.6 (0.7–3.9)	1.4 (0.6–3.3)^a^
	Suicide attempt (lifetime)	No IPV exposure	**3.1 (1.6**–**6.1)**	**2.3 (1.1**–**4.8)**	2.0 (0.9–4.3)
		IPV exposure	**1.6 (1.1**–**2.2)**	1.3 (0.9–1.9)	1.2 (0.8–1.7)^b^
	Suicidal ideation (past-year)	No IPV exposure	**3.8 (1.8**–**8.1)**	**3.1 (1.4**–**6.8)**	**2.6 (1.2**–**5.9)**
		IPV exposure	**1.5 (1.0**–**2.3)**	1.2 (0.8–1.9)	1.1 (0.7–1.7)^c^
	Non-suicidal self-harm (past-year)	No IPV exposure	**6.6 (2.4**–**19.2)**	**5.0 (1.7**–**15.2)**	**3.8 (1.2**–**11.9)**
		IPV exposure	1.7 (0.9–3.4)	1.3 (0.7–2.6)	1.2 (0.6–2.4)^d^
Number of distinct IPV perpetration behaviours	Suicide attempt (past-year)	No IPV exposure	2.0 (0.8–5.1)	1.6 (0.6–4.2)	1.3 (0.5–3.9)
		IPV exposure	**1.5 (1.0**–**2.2)**	1.3 (0.8–1.8)	1.2 (0.8–1.8)
	Suicide attempt (lifetime)	No IPV exposure	**1.7 (1.1**–**2.7)**	1.4 (0.9–2.3)	1.4 (0.8–2.2)
		IPV exposure	**1.3 (1.1**–**1.6)**	**1.2 (1.0**–**1.4)**	1.1 (0.9–1.4)
	Suicidal ideation (past-year)	No IPV exposure	**2.3 (1.5**–**3.7)**	**2.0 (1.2**–**3.4)**	**1.9 (1.1**–**3.2)**
		IPV exposure	**1.3 (1.0**–**1.6)**	1.1 (0.9–1.4)	1.1 (0.8–1.3)
	Non-suicidal self-harm (past-year)	No IPV exposure	**2.8 (1.6**–**5.0)**	**2.3 (1.3**–**4.2)**	**2.2 (1.2**–**3.9)**
		IPV exposure	**1.5 (1.1**–**2.1)**	1.3 (0.9–1.8)	1.2 (0.9–1.7)
Threat	Suicide attempt (past-year)	No IPV exposure	**9.8 (1.2**–**81.3)**	6.4 (0.7–57.7)	4.7 (0.6–35.4)
		IPV exposure	2.4 (0.9–6.5)	1.9 (0.7–4.9)	1.8 (0.7–4.7)
	Suicide attempt (lifetime)	No IPV exposure	**3.7 (1.3**–**10.5)**	2.4 (0.8–7.4)	2.1 (0.7–6.3)
		IPV exposure	**1.9 (1.1**–**3.1)**	1.6 (0.9–2.8)	1.5 (0.9–2.7)
	Suicidal ideation (past-year)	No IPV exposure	**4.5 (1.3**–**15.5)**	3.2 (0.9–11.9)	2.7 (0.7–10.1)
		IPV exposure	**2.2 (1.3**–**3.8)**	**1.9 (1.1**–**3.4)**	**1.8 (1.0**–**3.2)**
	Non-suicidal self-harm (past-year)	No IPV exposure	**7.3 (1.0**–**53.8)**	4.9 (0.6–36.7)	3.8 (0.6–26.6)
		IPV exposure	**3.0 (1.4**–**6.5)**	**2.3 (1.0**–**5.2)**	**2.3 (1.0**–**5.0)**
Pushing	Suicide attempt (past-year)	No IPV exposure	–	–	–
		IPV exposure	–	–	–
	Suicide attempt (lifetime)	No IPV exposure	2.2 (0.8–6.0)	1.5 (0.5–4.6)	1.5 (0.5–4.8)
		IPV exposure	1.3 (0.9–1.9)	1.0 (0.6–1.5)	0.9 (0.6–1.3)
	Suicidal ideation (past-year)	No IPV exposure	**4.9 (2.0**–**12.3)**	**3.9 (1.5**–**10.2)**	**3.7 (1.4**–**9.8)**
		IPV exposure	1.3 (0.8–2.1)	1.0 (0.6–1.7)	0.9 (0.5–1.5)
	Non-suicidal self-harm (past-year)	No IPV exposure	4.1 (0.6–27.3)	2.8 (0.4–17.1)	3.2 (0.5–19.4)
		IPV exposure	1.5 (0.7–3.1)	1.0 (0.5–2.2)	0.9 (0.4–2.0)
Kicking	Suicide attempt (past-year)	No IPV exposure	–	–	–
		IPV exposure	–	–	–
	Suicide attempt (lifetime)	No IPV exposure	3.1 (0.9–10.7)	2.2 (0.6–8.9)	2.1 (0.5–9.3)
		IPV exposure	**2.3 (1.5**–**3.4)**	**1.7 (1.1**–**2.6)**	**1.6 (1.0**–**2.5)**
	Suicidal ideation (past-year)	No IPV exposure	**5.5 (1.8**–**16.9)**	**4.4 (1.3**–**14.7)**	**4.0 (1.2**–**13.9)**
		IPV exposure	**1.6 (1.0**–**2.6)**	1.2 (0.7–2.0)	1.1 (0.6–1.8)
	Non-suicidal self-harm (past-year)	No IPV exposure	3.6 (0.7–17.9)	2.8 (0.5–16.8)	2.1 (0.4–12.6)
		IPV exposure	**2.2 (1.0**–**4.8)**	1.4 (0.7–3.1)	1.3 (0.6–2.8)
Forcing your partner to do something sexual	Suicide attempt (past-year)	No IPV exposure	5.0 (0.4–70.1)	4.8 (0.3–76.0)	2.2 (0.16–30.9)
		IPV exposure	**7.3 (1.1**–**50.2)**	**10.3 (2.0**–**53.7)**	**10.1 (1.5**–**66.0)**
	Suicide attempt (lifetime)	No IPV exposure	0.9 (0.1–7.6)	1.0 (0.1–9.5)	0.5 (0.1–4.9)
		IPV exposure	1.5 (0.5–4.9)	1.7 (0.5–5.7)	1.7 (0.5–5.9)
	Suicidal ideation (past-year)	No IPV exposure	1.4 (0.2–11.8)	1.4 (0.1–13.1)	0.8 (0.1–6.6)
		IPV exposure	0.6 (0.1–3.4)	0.7 (0.1–3.1)	0.6 (0.1–2.9)
	Non-suicidal self-harm (past-year)	No IPV exposure	**13.4 (2.4**–**75.8)**	**12.0 (2.1**–**69.8)**	**5.8 (1.0**–**33.2)**
		IPV exposure	5.0 (0.2–114.1)	6.4 (0.3–125.7)	6.7 (0.4–109.6)

*Note*: Model 1: adjusted for gender – age – ethnicity – marital status – tenure and area deprivation. Model 2: adjusted for model 1 variables – and also for other life adversities. Model 3: adjusted for model 2 variables – and also for hazardous alcohol use – and drug use in the past year. Bold text indicates statistical significance.

a IPV exposure IT: 0.6 (95% CI 0.1–4.2) – *p* = 0.604.

b IPV exposure IT: 0.6 (95% CI 0.3–1.4) – *p* = 0.217.

c IPV exposure IT: 0.4 (95% CI 0.2–1.0) – *p* = 0.059.

d IPV exposure IT: 0.3 (95% CI 0.1–1.2) – *p* = 0.081.

## Discussion

### Summary of findings

Lifetime IPV perpetration, number of IPV perpetration behaviours and the separate IPV behaviour indicators were each associated with past-year and lifetime suicide attempt, past-year suicidal ideation, and past-year non-suicidal self-harm, after adjustment for sociodemographic and socioeconomic explanatory variables. Associations were evident in men and women separately, with no evidence for interaction by gender. Further adjustment for other (non-IPV-related) life adversity, hazardous alcohol use, drug use and IPV victimization substantially attenuated most associations (objective 1). Estimates for the association between IPV perpetration and suicide attempt, suicidal ideation, and non-suicidal self-harm were generally higher among participants without lifetime IPV victimization, although we did not find statistical evidence for interaction (objective 2).

### Interpretation and previous findings

The association between IPV perpetration and suicidality is evident in the general population, where many will not be engaged with criminal justice or health/social care services. The association is largely accounted for by shared risk factors for both suicidality and IPV perpetration, including non-IPV life adversity, drug/alcohol misuse and IPV victimization. While we did not formally assess causality, our results are consistent with a link between psychiatric disorders and IPV perpetration (Oram *et al.*, 2013; Yu *et al.*, 2019). Extending our previous work on IPV perpetration and mental health service use (Bhavsar *et al.*, 2023), these results indicate that IPV perpetration, as well as IPV victimization, may be a clinically relevant feature of mental health encounters in crisis services.

It has been proposed that ‘bi-directional’ IPV, where both partners use and experience violence within a relationship (Machado *et al.*, 2024), represents a qualitatively different phenomenon compared to unidirectional and asymmetrical situations. Our results imply that the association of IPV perpetration with suicidality is evident even among those who do not report IPV victimization (i.e., unidirectional IPV) and, indeed, may be stronger among this group. Further research should consider psychological factors that may explain greater suicidality experienced by perpetrators of IPV, some of which may contribute to stronger associations in unidirectional perpetrators of IPV compared to bi-directional perpetrators of IPV. For example, unidirectional IPV perpetrators may feel greater shame about perpetrating harm towards one’s partner, or conflict of one’s behaviour with one’s moral code, or greater rejection by others, compared to perpetrators of bi-directional IPV (although these mechanisms may also be relevant to suicidality in perpetrators of bi-directional IPV). Given that the elevated odds of suicidality were attributable to other life adversities, our results suggest that IPV perpetration may be related to the experience of multiple adversities, and that these multiple adversities may, in turn, increase suicide risk.

Few studies have explored IPV perpetration and suicidality in the general population data, particularly in high-income settings. A UK study found evidence for crude associations between perpetrating dating violence and self-harm in 16-year-old male and female participants (Herbert *et al.,* 2021), consistent with but slightly smaller than our estimates. However, it focused only on birth cohort participants and did not evaluate explanatory variables. Global studies also predominantly focus on younger populations, with mixed findings regarding gender differences in the association between IPV perpetration and suicidality (Verduin *et al.*, 2013; Ulloa and Hammett, 2016; Stark *et al.*, 2020).

Verduin *et al.* (2013) examined suicidal ideation in victims and perpetrators of IPV in a Rwandan general population survey, but only analysed coupled individuals and reported gender-adjusted estimates only, conflating results from men and women. They found that the OR for suicidal ideation comparing participants reporting to be both victim and perpetrator compared to others was 1.5 (0.6–3.9). No participants in this study reported themselves to be only perpetrators of IPV. A US study focusing on 18–34-year-olds only found that past-year IPV status (non-violent, perpetrator-only, victim-only, bidirectional) was associated with suicidal ideation, but not suicide attempt (Ulloa and Hammett, 2016). Bi-directional violence had the highest prevalence of suicidal ideation, while the perpetrator-only group had higher prevalences compared to the victim-only group. A Nigerian study of 13–24-year-olds (Stark *et al.*, 2020) found that in females, and this remained statistically significant after adjusting for IPV exposure, in contrast to our findings. This may be suggestive of an influence of age on the strength of associations between IPV perpetration and suicidal ideation in women, with stronger associations in younger age groups. In men, the association was non-significant and was further attenuated after adjustment. In contrast to Stark *et al.* (2020), a Ugandan study of the same age group found a significant association between IPV perpetration and suicidal ideation in males, including after adjustment for IPV exposure, but not females (Cohen et al., 2022). Again, this contrasts our findings, which suggest that after adjustment for IPV exposure, the association between suicidal ideation and lifetime IPV perpetration is non-significant for both genders, and in contrast to Stark *et al.* (2020), suggests that the association is stronger in younger men, rather than younger women. Therefore, whether IPV perpetration is a stronger risk marker for suicide in women compared to men requires further investigation in age-representative samples. While we found no statistical interaction by gender of the association of IPV perpetration with suicidality, evidence strongly suggests distinct differences in IPV and suicidality between men and women, and future research should consider the possibility of gender-specific mechanisms underlying this association, in gender-disaggregated analyses.

### Strengths and limitations

In contrast to previous research focusing on those in contact with criminal justice or health services or with IPV perpetrator programmes, our study presents the first examination of the association between IPV perpetration and suicidality in a recent UK general population sample, which can contribute to the development of a national picture of this association and inform population-level strategies to address both suicide (Department of Health and Social Care, 2023) and IPV perpetration (Hester *et al.*, 2020). With the exception of perpetration of sexual IPV (where small numbers reporting this meant estimates were unstable), associations were successfully estimated, and we were able to explore the impact of adjustment for various risk factors, which led to the attenuation of nearly all associations. This suggests that the experience of multiple adversities, together with IPV perpetration, poses a suicide risk.

Our study has limitations. The data analysed were cross-sectional, preventing temporal and causal inference. While we included covariates based on theoretical knowledge, some relevant factors may have been omitted and/or remain unknown. IPV is a broad construct containing multiple domains of harm occurring both as temporally separate incidents and as periods of exposure to harm incurred over time (Oram *et al.*, 2022). Items for IPV perpetration analysed in this paper did not measure coercive control, financial abuse, online or technology-assisted harm, the severity or frequency of harm, or patterns of escalation, which are all important aspects of IPV (Hamby, 2014). This limits the generalizability of our findings. Some IPV harmful behaviour not analysed in this paper may display different patterns of association with suicidality. For example, financial IPV may be more strongly related to suicide because of underlying economic food insecurity, which is in turn related to suicidality (Kaggwa *et al.*, 2023). Our analyses were disaggregated by gender *a priori*, to allow assessment of the results for men and women separately. Nevertheless, any direct comparison of descriptive data on the prevalence of IPV perpetration and different types of harmful behaviour in men and women should be done with caution, because of the incomplete measurement of IPV perpetration in our data, and the possibility of differential misreporting. Both perpetration of IPV and suicidality are considered socially undesirable in some contexts, and may have been vulnerable to under-reporting (Freeman *et al.*, 2015). This could have resulted in biased estimates given any differential misclassification, for example, if those reporting IPV perpetration were less likely to disclose a suicide attempt if it had occurred. We know of no evidence on how and whether IPV perpetration influences disclosure of suicidality or vice versa. Missing data could have biased our results if the association of interest was negative or null among those with missing data, for example, on IPV perpetration and suicidality.

### Implications for practice

Perpetration of IPV is an epidemiological marker of suicide risk in men and women, even in those who have not been victims of IPV. Efforts to improve clinical responses to the perpetration of IPV in people with psychiatric conditions, particularly those who have the experience of other adversities, through strengthened identification, assessment and management practices, including being aware of and sensitive to the risk of suicide, could reduce suicidality and self-harm in this population. Services encountering people who have perpetrated IPV, such as perpetrator programmes and police/probation services, could benefit from specific training in the identification and management of suicide risk in this group. Behaviour change programmes for perpetrators of IPV could be refined to place greater emphasis on suicide prevention and harm reduction, informed by our findings. Our results also highlight the need for evidence-based interventions to reduce suicidality in people perpetrating IPV who are in contact with mental health services.

### Implications for research

Our results highlight the overall association of IPVP with suicidal behaviours, but assume similar associations across sociodemographic groups. Future research, for example, generating adequately powered evidence on differences in these associations based on age or ethnic group, could inform targeted prevention/intervention strategies. Future work assessing the impact of increasing severity, or frequency, of IPV perpetration on risk of suicidality could also be helpful in informing future intervention strategies. Finally, further work should also consider the relevance of suicidality to a variety of harmful behaviours perpetrated within IPV. There remains limited evidence for interventions to reduce suicidality for perpetrators of IPV, including perpetrators who are also IPV victims. A recent review of interventions for suicidality among victims of IPV found limited and mixed evidence (Jiwatram-Negrón *et al.*, 2023), indicating the need to develop interventions to address suicidality for both victims and perpetrators of IPV. In this context, our results suggest that developing interventions for people presenting to services with both IPV perpetration and suicidality may require attention to shared risk factors, including life adversity, IPV exposure and substance misuse.

## Conclusions

Perpetration of IPV is associated with suicide attempt, suicidal ideation and non-suicidal self-harm in the English household population. These associations are accounted for by life adversities, IPV victimization and substance use. Targeted identification and support for perpetrators of IPV could positively impact responses to suicidality and non-suicidal self-harm.

## Data Availability

Requests for access to the 2014 dataset should be made to the Data Access Request Service at NHS Digital. Code availability: Survey data collection was programmed in Blaise, a computer-assisted interviewing system and survey processing tool for the Windows operating system. The system is developed by Statistics Netherlands and has been designed for use in official statistics. It is available to National Statistical Institutes and related research institutes. Data management and analysis was conducted in Stata 17, and syntax is available from the authors on request.
